# The Trial within Cohorts (TwiCs) study design in oncology: experience and methodological reflections

**DOI:** 10.1186/s12874-023-01941-5

**Published:** 2023-05-13

**Authors:** Rob Kessels, Anne M. May, Miriam Koopman, Kit C. B. Roes

**Affiliations:** 1 Dutch Oncology Research Platform, Netherlands Cancer Institute, Amsterdam, the Netherlands; 2 Julius Center for Health Sciences and Primary Care, University Medical Centre Utrecht, Utrecht University, STR 6.131 , P.O. Box 85500, 3508 GA Utrecht, the Netherlands; 3 Department of Medical Oncology, University Medical Centre Utrecht, Utrecht University, Utrecht, the Netherlands; 4 Department of Health Evidence, Radboud University Medical Center, Section Biostatistics, Nijmegen, the Netherlands

**Keywords:** Trials within Cohorts, TwiCs, Cohort multiple randomized controlled trial, Oncology, Non-compliance, Efficacy estimand

## Abstract

A Trial within Cohorts (TwiCs) study design is a trial design that uses the infrastructure of an observational cohort study to initiate a randomized trial. Upon cohort enrollment, the participants provide consent for being randomized in future studies without being informed. Once a new treatment is available, eligible cohort participants are randomly assigned to the treatment or standard of care. Patients randomized to the treatment arm are offered the new treatment, which they can choose to refuse. Patients who refuse will receive standard of care instead. Patients randomized to the standard of care arm receive no information about the trial and continue receiving standard of care as part of the cohort study. Standard cohort measures are used for outcome comparisons. The TwiCs study design aims to overcome some issues encountered in standard Randomized Controlled Trials (RCTs). An example of an issue in standard RCTs is the slow patient accrual. A TwiCs study aims to improve this by selecting patients using a cohort and only offering the intervention to patients in the intervention arm. In oncology, the TwiCs study design has gained increasing interest during the last decade. Despite its potential advantages over RCTs, the TwiCs study design has several methodological challenges that need careful consideration when planning a TwiCs study. In this article, we focus on these challenges and reflect on them using experiences from TwiCs studies initiated in oncology. Important methodological challenges that are discussed are the timing of randomization, the issue of non-compliance (refusal) after randomization in the intervention arm, and the definition of the intention-to-treat effect in a TwiCs study and how this effect is related to its counterpart in standard RCTs.

## Introduction

Randomized Controlled Trials (RCTs) are generally considered the golden standard in experimental design for evaluating the efficacy of medical treatments. Random allocation of patients to different treatment groups is expected to create prognostically comparable groups, where the only difference between groups is the assigned treatment, thus minimizing the different sources of bias. Currently, the standard approach is to conduct separate randomized clinical trials, each investigating the effect of a single intervention in a single disease. However, it has been argued that this ‘classical’ way has become challenging due to several reasons, such as uncompleted trials, high drop-out rate in the control group and the limited external validity. These three reasons will be discussed below.

First, it has been argued that standard RCTs in oncology often remain uncompleted because they are confronted with poor and slow accrual of patients, high costs, and inadequate funding [1]. Slow accrual might be due to the fact that only a small fraction of all cancer patients are actually enrolled in a trial, *e.g.* patients refuse to be randomized. Also, due to an increased heterogeneity in tumor types, the number of small subgroups increases. This leads to trials where only a small subset of patients is eligible, making accrual even more challenging.

Second, the issue of high drop-out in the control group might be due to the experience of disappointment of patients who are randomized to a control treatment [2]. This is especially true for open-label trials that cannot be performed in a blinded setting. Many oncology RCTs are more likely to be open-label trials [3], because it is not uncommon to compare two arms that only differ in the way the treatment is administrated. As a consequence, one could expect differential drop-out across the treatment arms.

Third, standard RCTs have been criticized for their limited external validity, because participants selected in RCTs are generally not representative of the general population [4]. Clinical trials tend to exclude elderly patients and/or patients with common comorbidities [5], and phase 3 clinical trials often fall short of including a representative number of patients from diverse racial and ethnic groups [6]. Also, RCTs generally implement many eligibility criteria which diverge from the traditional disease definition. Hence, RCTs are prone to selection bias. External validity is also affected if the RCT does not mimic routine clinical practice. Participating in a trial often involves more frequent and closer monitoring of patients compared to routine clinical practice, and this might lead to different results observed in trials compared to clinical practice. For example, progression of the disease might be detected earlier in trials or the patients report improvement in their quality of life during trial participation due to the close attention that is given to them by trial staff. These external validity issues lead to concerns regarding the generalizability of results. Pragmatic trials aim to (partially) solve these external validity issues by retaining the randomization component and adopting characteristics of routine clinical practice [7]. However, as pragmatic RCTs are still ‘classic’ RCTs, they may face potential disadvantages like poor accrual and risk of drop-outs.

The abovementioned problems of standard RCTs raised interest for methodological innovation. The Trial within Cohorts (TwiCs) study design was developed to address some of the issues encountered in standard RCTs. The TwiCs design was originally proposed by Relton et al. [8], who introduced it as the ‘cohort multiple randomized controlled trial’. A TwiCs study is performed within an prospective observational cohort study. In a prospective observational cohort study, a group of patients with the condition of interest receiving Standard of Care (SOC), is followed over time. In this prospective cohort, broad eligibility criteria are often used, where all patients sharing a certain disease are eligible. For example, in the Prospective ColoRectal Cancer Cohort (PLCRC) [9, 10] all Dutch patients diagnosed with a malignancy in the colon and/or rectum and patients with bowel or anal cancer are eligible and in the Utrecht cohort for multiple breast cancer intervention studies and long-term evaluation (UMBRELLA) cohort [11], all patients with invasive breast cancer and ductal carcinoma in situ are eligible. These broad eligibility criteria for cohort enrollment ensure that the cohort study is a good representation of the population. Upon cohort enrollment, patients give informed consent for cohort participation, after which data on clinical and patient reported outcomes are regularly collected at baseline and during follow-up. Additionally, patients are asked for informed consent to be randomized in future RCTs within the cohort. Patients are informed that they will be offered an alternative treatment if they are randomly selected for the intervention group. They are also informed that they will not be contacted when randomly selected for the control group and that their data will be used in a trial context. Once an alternative treatment becomes available, an RCT is designed and eligible patients within the cohort are identified. One of the trial eligibility criteria is whether cohort participants provided informed consent for randomization in future RCTs. Of these eligible participants, a random selection will be invited to undergo the new treatment or intervention. These participants receive a new informed consent providing them with all information available of the alternative treatment. Eligible participants who were not randomly selected for the intervention group are randomly selected for the control group, receive SOC, and are not informed about the trial (and hence receive no additional informed consent). This informed consent procedure was introduced as the two staged-informed consent [12]. At the end of the trial, a third stage was proposed in which all cohort participants, irrespective of their specific trial participation, receive the trial results (*e.g.* in an annual newsletter). The staged-informed consent overcomes that participants are randomized without their prior consent. This mitigates ethical concerns, however, caution is warranted due to interpretation differences between ethics committees [13]**.** The understanding of the staged-informed consent procedure was evaluated among patients participating in oncology TwiCs studies and it was found that patients did not have ethical objections to serve as control without further notice [14, 15]**.**

The rationale behind the TwiCs study design is that it prevents (possible) patient drop-out and contamination in the control group due to lack of information about the trial, thus reducing patient disappointment in missing an opportunity for a new alternative treatment. This design element also resembles more closely the informed consent procedure used in routine clinical practice, as patients are usually not informed about treatments they cannot receive [8]. Moreover, because a TwiCs study uses the infrastructure of an observational cohort study, it can improve accrual rate and accrual speed as eligible patients are already known and can therefore be approached easily. In addition, the availability of an observational cohort infrastructure makes it possible to base eligibility on information from routine cohort measurements (*e.g.* in the UMBRELLA Fit trial [16], patients were invited for an exercise intervention study based on cohort data and not based on the physicians estimation whether the patient would be able to do the exercises). When adopting such a selection procedure, it may ensure that selection of patients in a TwiCs study is affected less by the preference of a physician, thus reducing selection bias [17]. This improves representativeness and the generalizability of the trial results. It is important to mention that it is crucial that the SOC follow-up schedule matches the purpose and the research question of the TwiCs study, as it is undesirable or even impossible to adapt the SOC follow-up schedule.

Because a TwiCs study is performed within an observational cohort study, it brings in another possible advantage: performing multiple RCTs over time using patients of the same cohort (from where it owes its original name ‘cohort *multiple* RCT’). This enables the investigation of multiple treatments within the same patient group. This capacity is aligned with the recent development of master protocols to study multiple therapies for a single disease, a single therapy for multiple diseases, or both [18], which have been initiated for the study of cancer therapies [19]. Woodcock and LaVange [18] discuss three master protocol studies: the umbrella trial, the basket trial and the platform trial. In an umbrella trial, multiple therapies are tested in the context of a single disease, whereas in a basket trial, a single therapy is tested in the context of multiple diseases. In a platform trial, multiple therapies are studied in the context of a single disease in a perpetual manner: therapies are allowed to leave or enter the platform based on a decision algorithm. Master protocol studies were also developed to face the increasing costs and other challenges of standard RCTs, such as poor accrual due to precision medicines only suited for a small subset of patients. The availability of an observational cohort where enrolled patients are all diagnosed with the same disease enables the execution of an umbrella or platform trial using a master protocol. A TwiCs study can be placed in the context of a platform trial if multiple TwiCs studies are performed to study the effect of multiple alternative treatments for treating the disease of interest. However, for a TwiCs study, the basis is a broad observational cohort that can be used to initiate multiple stand-alone trials each specified in a separate protocol, each answering a different research question and the tested treatments are not necessarily related. Moreover, these TwiCs studies are not known in advance when starting the cohort study. In contrast, a platform trial investigates the effect of multiple alternative treatments according to a predefined plan described in one single master protocol and these treatments are part of a series of related treatments.

Given the assumed advantages of TwiCs studies, TwiCs studies have gained interest in several research fields such as psychosocial and rehabilitation interventions [20], interventions for mental illness [21], and depression treated by homeopaths [22]. In this article, we focus on the application of TwiCs designs within the oncology setting. Specifically, the TwiCs design is entailed with several methodological challenges of which we discuss the implications with respect to the applicability of TwiCs studies for (future) oncology trials, using experiences from previous TwiCs studies in oncology. In this discussion, we pay attention to the question whether a TwiCs study is able to answer the same research question that is usually answered by performing a standard RCT. In addition to methodological challenges, the TwiCs design also faces several ethical issues [13, 23]. Because the focus of this article is on the methodological challenges, these ethical issues are not discussed here. The article is structured as follows: in the next section, we provide an overview of examples of TwiCs studies within the oncology setting. Then, we discuss the methodological challenges faced in TwiCs studies and explain how these challenges can be dealt with in oncology TwiCs studies. This article ends with a discussion concerning future considerations.

## Overview of (applied) TwiCs in the oncological setting

Within the oncology setting, there are several examples of initiated TwiCs studies. These studies are described in Table 1. These trials were or are still conducted within The Netherlands, with exception of the TILT trial, which was conducted in the UK. Five trials (TILT, RECTAL BOOST, UMBRELLA FIT, SPONGE and VERTICAL) are now completed, while two trials are still ongoing. The TILT trial is the only (and first) trial that used a TwiCs design to study the effect of an investigational medicinal product. It is a feasibility study aiming to verify the feasibility of performing a randomized trial of intra-pleural bacterial immunotherapy using a TwiCs design with the pre-specified recruitment, attrition, and data completeness as primary outcome measures.

**Table 1:** Overview of applied TwiCs studies within the oncological setting

Trial	Description	Status	Planned number of patients
TILT [24, 25]	Feasibility trial of an investigational medicinal product to treat mesothelioma. The aim of this study was to answer whether a full-scale version of the trial is possible and whether a TwiCs study is appropriate for mesothelioma trials. The investigational product is called OK-432 (“dead” bacteria) which is used to stimulate immune cells to attack the mesothelioma. The trial was initiated within the ASSESS-meso cohort [26]	Completed	45 patients
RECTAL BOOST [27–29]	Randomized controlled trial for pre-operative dose-escalation BOOST in locally advanced rectal cancer. The trial was originally initiated within the prospective data collection initiative on colorectal cancer (PICNIC) cohort, which has been renamed to PLCRC [9, 10]	Completed	120 patients
HONEY [30]	Clinical trial of assessing the effect of hyperbaric oxygen therapy in breast cancer patients with late radiation toxicity. The trial is initiated within the UMBRELLA cohort [11]	Ongoing	120 patients
UMBRELLA FIT [16, 17, 31]	Clinical trial investigating the effect of an exercise program on the quality of life of patients with breast cancer. The trial was initiated within the UMBRELLA cohort [11]	Completed	192 patients
MEDOCC-CrEATE [32]	Clinical trial investigating the effect of circulating tumor DNA guided adjuvant chemotherapy in stage II colon cancer. The presence of circulating tumor DNA is only assessed in the alternative treatment group. The trial is initiated within the PLCRC [9, 10]	Ongoing	60 patients with circulating tumor DNA. In total, 1320 patients
SPONGE [33, 34]	Clinical trial investigating the impact of retractor SPONGE-assisted laparoscopic surgery on duration of hospital stay and postoperative complications in patients with colorectal cancer. The trial is initiated within the PLCRC [9, 10] and is a follow-up trial of the RECTAL BOOST trial. Patients of the RECTAL BOOST trial were also eligible for the SPONGE trial	Completed	196 patients
VERTICAL [35–38]	Clinical trial comparing conventional radiotherapy with stereotactic body radiotherapy in patients with spinal metastases. The trial is initiated within the prospective evaluation of interventional studies on bone metastases (PRESENT) cohort [39]	Completed	110 patients

In all evaluated trials, the experimental group is offered an alternative treatment/intervention, while the control group receives SOC. In the MEDOCC-CrEATE trial, a somewhat different study procedure is undertaken. This trial is conducted within the PLCRC, an observational cohort study for patients diagnosed with colorectal cancer. The MEDOCC-CrEATE trial investigates the willingness of patients to receive adjuvant chemotherapy after detection of circulating tumor DNA, and to assess the effect of circulating tumor DNA guided adjuvant chemotherapy. More specifically, approximately one week after surgery, eligible patients (who also provided consent to be randomized in future trials) for the MEDOCC-CrEATE trial are randomized to the intervention or control arm. Following the TwiCs study design, only patients randomized to the intervention arm are asked informed consent for the immediate analysis of their circulating tumor DNA status of a post-surgery blood sample. Patients who have detectable circulating tumor DNA in their blood are offered adjuvant chemotherapy, which they can either accept or refuse. The patients who refuse receive control treatment, which consists of routine post-surgery follow-up care. The patients without detectable circulating tumor DNA in their blood (as well as patients who provide no informed consent for the immediate analysis of circulating tumor DNA) receive control treatment. Patients randomized to the control group receive no information about the trial, and their post-surgery blood samples are not tested immediately for circulating tumor DNA. The difference with regular TwiCs studies is that in the MEDOCC-CrEATE trial, patients are not directly randomized for a prospective alternative treatment, but they are primarily randomized to the chance to find out whether they have circulating tumor DNA in their blood, which in turn determines whether they are offered an alternative treatment.

In addition to the trials and related cohorts listed in Table 1, more cohorts have been set-up where the TwiCs design has been introduced. For example, in the Netherlands cohort studies for bladder cancer [40], gastro-intestinal cancer [41], pancreatic cancer [42], and prostate cancer [43] have been initiated, in which TwiCs studies can be embedded. In the UK, bladder cancer [44] and prostate cancer [45] cohorts have been started. In the UK prostate cancer cohort, researchers performed a pilot study to verify if patients are willing to participate in a cohort study and what is their opinion on the staged-informed consent [45]. In addition, in the prostate cancer setting, the TwiCs design was mentioned as promising trial design to solve recruitment issues when comparing focal therapy to active surveillance, radical therapy, or prostatectomy in a randomized setting [46–49].

## Methodological considerations

### Timing of randomization

An important element in designing a TwiCs study is the timing of randomization, which varies according to the intervention or treatment under study [50]. Cohort participants can be randomized to the control or intervention arm at one moment in time, which is a feasible approach in a closed or recruiting cohort, and is referred to as the ‘single-batch sampling approach’. An alternative to the single-batch sampling approach is the ‘multiple-batch sampling’ approach [50], where a subgroup of cohort participants is randomized at one moment in time. In this approach the cohort continues to randomize eligible patients who are not allocated yet to the control or intervention arm. This approach is also feasible for closed or recruiting cohorts. Multiple rounds of randomization are conducted within the cohort. This approach was applied in the UMBRELLA FIT trial [16, 17, 31] and is also adopted in the HONEY trial [30].

For some interventions, the single and multiple-batch randomizations are not feasible, because screening for trial eligibility and randomization needs to take place within a short timeframe right after diagnosis, progression or relapse [50]. This entails that eligible patients should be randomized as soon as they consented to the trial, which makes it impossible to randomize all patients at the same time. This randomization procedure is comparable to the way patients are randomized in standard RCTs. Within a cohort setting, the randomization approach often requires a recruiting cohort and can be applied shortly after the start of the cohort. The latter implies that upon (cancer) diagnosis, patients are invited to participate in a cohort study where a cohort consent and possible consent to randomization into future RCTs are provided (two staged-informed consent procedure). In case the intervention or treatment needs to be administered shortly after diagnosis, eligible patients for the trial are randomized immediately or very soon after cohort enrollment. In these situations, it is impossible to leave much time between cohort enrollment and the moment patients are randomized into a TwiCs study. This procedure was applied in the RECTAL BOOST trial [27–29], where patients provided informed consent for cohort enrollment after being diagnosed with locally advanced rectal cancer. Directly after cohort enrollment, patients who consented for randomization into future RCTs (among other trial eligibility criteria) were randomized to the control arm or to the alternative treatment arm. Patients in the control arm received standard chemoradiation and patients randomized to the alternative treatment arm were offered a boost before chemoradiation. By nature of the design, patients in the control arm were not informed about this boost possibility. The same procedure was used in the VERTICAL trial [35–38].

When randomization into a TwiCs study starts at the same day or shortly after cohort enrollment, it is inevitable that the ‘future’ trial is already known by researchers upon the moment that patients sign the two staged-informed consent. This may still lead to selection bias in the trial, which is exactly what one wants to minimize when conducting a TwiCs study. Furthermore, this potential selection bias into the trial brings in another possible risk—selection into the trial may trickle down to selection for cohort enrollment and thus representativeness of the cohort. When a newly diagnosed cancer patient is suited for cohort enrollment, but ineligible for the TwiCs study upon diagnosis, it is highly undesirable to exclude that patient from the cohort. In other words, when recruiting patients for the cohort study, eligibility criteria for future RCTs should not be considered. This risk plays a potential role when the TwiCs study investigates the effect of (new) interventions of which it is known that these interventions start shortly after cohort enrollment. The advantages of TwiCs studies over standard RCTs (*e.g.* fast accrual) should not tempt researchers to start a cohort study for the sake of a clinical trial as this would slowly turn the trial into an RCT following the controversial Zelen design, where patients are randomized before consent is given [51].

### Non-compliance in the alternative treatment arm

In a TwiCs study, only patients randomly selected for the alternative treatment arm are asked to provide informed consent *after* randomization (but *before* treatment). As stated previously, patients randomly selected for the control arm receiving SOC are not notified about the trial, are therefore not asked informed consent and are not aware of the alternative intervention. As a consequence, only patients randomized to the alternative treatment arm can refuse this treatment (*after* randomization), and patients who refuse will receive SOC. This will lead to non-compliance in the alternative treatment arm. Since control patients are not informed about the trial, it is highly unlikely that this type of non-compliance (refusal of assigned treatment) is randomly distributed over study arms (as opposed to standard RCTs). It is important to consider this selected non-compliance when defining the research question, determining the effect size and calculating the required sample size of TwiCs studies. In the remainder of this manuscript, non-compliance is defined as refusal of an alternative treatment or intervention (if offered) after randomization.

Most oncology TwiCs studies presented in Table 1 anticipated on the occurrence of non-compliance in the treatment arm during the design phase by including the expected non-compliance rate in the sample size calculations. However, it is worth mentioning that the anticipated non-compliance rate might deviate from the actual non-compliance rate. For example, in the UMBRELLA FIT trial, the anticipated non-compliance rate was 30%, but after 152 patients of the initially required 166 patients were recruited, the actual non-compliance rate was 45% [17]. In the RECTAL-BOOST trial, there was an overall non-compliance rate of 27% compared to an expected rate of 20% [28]. In the VERTICAL trial, the assumed non-compliance rate was 10% while the actual rate was 27% [36]. As the TILT trial was a feasibility study, non-compliance rate in the alternative treatment arm was considered a primary outcome measure, but the authors also included failure to complete follow-up in the control arm in the non-compliance definition, which is why in the TILT trial the non-compliance rate definition was different compared to the other trials [25]. The study was considered feasible with respect to the non-compliance rate if that rate was below 10%. Of the 12 randomized patients, one patient in the alternative treatment arm refused the treatment after randomization and one control patient did not complete the follow-up schedule, which indicates that the 10% maximum was exceeded.

These results show that the actual non-compliance rates deviate from the expected non-compliance rate. The non-compliance rate in the treatment arm can be interpreted as a methodological challenge of a TwiCs study that requires careful consideration when defining the research question, the clinical endpoints and the determination the required sample size. In the upcoming subsections we will discuss the implications of non-compliance for the treatment effect estimate and the statistical power. However, before these aspects are discussed, it is necessary to first clarify which effect is estimated in a TwiCs trial and how this is connected to the research question. This will be the topic of the next subsection.

### Defining the efficacy estimand in a TwiCs study

For this discussion we consider the guidelines outlined in the ICH E9 (R1) draft addendum on “Estimands and Sensitivity Analysis in Clinical Trials” [52]. The estimand of a clinical trial can be defined as the targeted treatment effect that reflects the research question which is given by the research objective. It provides a summary at the level of the population of what the treatment effect would be in the same patients under different treatment options being compared. How the estimand is to be estimated should be specified in advance of the trial and once this is defined, the trial can be designed as such that it is possible to generate a reliable estimation of that treatment effect. For the definition of the estimand in a clinical trial, it is required to anticipate on so-called intercurrent events, which are defined as events that mark a change in the course of treatment and that influence the estimation and interpretation of treatment effects. Intercurrent events need to be addressed *a-priori* when describing the clinical research question of interest. In a TwiCs trial, non-compliance, or refusal of the alternative treatment *after* randomization but *before* started treatment, can be regarded as such an intercurrent event. It is obvious that this phenomenon will alter the interpretation of the treatment effect and should be considered when defining the estimand. More specifically, we should question what is the estimand in a TwiCs study, which effect is of interest (what is the research question?) and how can we estimate that effect. For the remainder of this discussion, we only consider the refusal of an offered alternative treatment or intervention after randomization as known intercurrent event in a TwiCs study and therefore only discuss the implication of that particular event.

First, it is important to assume that non-compliance due to refusal only occurs in the alternative treatment arm, which means that the intercurrent event is dependent on the assigned treatment. Second, it is also assumed that the occurrence of non-compliance will affect the treatment effect indefinitely—once a patient refuses offered treatment, the patient will receive the SOC for the remainder of the trial duration. Finally, it is assumed that the control patients do not have access and will not get the alternative treatment since these patients are not informed about the trial. In other words, there is no contamination in the control group.

#### Treatment policy strategy

The way non-compliance is addressed in the trial defines the research question that a TwiCs study is able to answer. One of the strategies to address the research question described in the ICH E9 (R1) draft guidance document is the treatment policy strategy—the intercurrent event is taken to be part of the treatment regimen of interest. The treatment effect is then estimated irrespective of the occurrence of an intercurrent event and the estimand is a combined effect of the initial randomized treatment and the treatment modified by the intercurrent event. Adopting a treatment policy approach has become known as the “Intention-to-treat” (ITT) approach—all patients are analyzed ‘as randomized’ regardless of the occurrence of the intercurrent event. For a TwiCs study, this implies that the non-compliance rate in the alternative treatment group is considered as part of the treatments being compared.

What does this mean for the interpretation of the ITT effect in a TwiCs study? This question can be answered by first taking a closer look at the ITT definition in a standard RCT. The ITT effect in a standard RCT is generally interpreted as the average causal effect (ACE) of the assigned treatment. The ACE measures the difference in the mean outcome between patients assigned to the alternative intervention and patients assigned to SOC. It has been argued that the ACE of a standard RCT is, on average, an unbiased estimate of the population mean effect of the alternative treatment compared to SOC in patients receiving treatment, under the assumption that treatments are randomly assigned, thereby assuming no confounding exists [53, 54]. Assuming that all patients also receive the assigned treatment, we refer to the ITT effect in a standard RCT as the ACE of received treatment for the remainder of this discussion. Although this technically is not the pure ITT definition (analyzing ‘as randomized’ regardless of taking up treatment), it is important to mention this nuance here when discussing the difference between the ITT definition of a standard RCT and a TwiCs study. In a TwiCs study, patients are also assigned a treatment, but the difference is that patients are offered alternative treatment when assigned to that treatment, whereas in a standard RCT we expect all patients to receive the assigned treatment. Therefore, to distinguish between the ACE of a standard RCT and a TwiCs study, we refer to received treatment and offered treatment, respectively.

Non-compliance is known to be a methodological problem that can lead to bias in estimating the ACE of received treatment in randomized experiments [55]. However, in a standard RCT, the refusal of treatment happens generally before randomization and these patients do not enter the trial. Furthermore, (potential) non-compliance is randomly distributed over the treatment arms in a standard RCT, which avoids immediate bias in estimating the ACE of a received treatment. In fact, only selective non-compliance in a standard RCT might lead to a more-or-less biased ACE of received treatment relative to the population value of the ACE of received treatment.

In contrast, for a TwiCs study, it is already expected beforehand that non-compliance only occurs in the alternative treatment arm *after* randomization; the intercurrent event occurs by nature of the design. As a result, non-compliance is known to be not random (selective non-compliance) and therefore, the treatment effect under the ITT-principle will be diluted when incorporating non-compliant patients. One might argue that non-compliance in a TwiCs study leads to a biased ACE of received treatment, but this is incorrect, because a TwiCs study simply adopts a different estimand compared to a standard RCT. As stated earlier, the ITT effect of a TwiCs study is the ACE of offered treatment rather than received treatment. This also means that when we speak of bias in a TwiCs study, it is important to refer to bias in the estimand of a TwiCs study. In a situation where the refusal rate of the alternative intervention in the trial matches that of the population, the ACE in a TwiCs study will provide an unbiased estimate of the population mean effect of the alternative intervention compared to SOC in patients who are offered the alternative intervention compared to patients receiving SOC [54]. The key point here is that a TwiCs study and a standard RCT estimate a different ITT effect (estimand) under the treatment policy strategy and therefore answer different research questions. Bias in a standard RCT is defined as bias relative to the effect of received treatment, whereas bias in a TwiCs study is defined as bias relative to the effect of offered treatment. Consequently, a TwiCs study will not provide a biased estimate of the ACE observed in a standard RCT, as sometimes falsely claimed (see the section on ‘Analysis of a TwiCs study’).

In the UMBRELLA FIT-, the RECTAL BOOST-, the VERTICAL-, and SPONGE trial, the primary analysis was done according to the ITT principle. However, as explained above, interpreting the ITT effect of a TwiCs study cannot be separated from the non-compliance rate in the alternative treatment arm. Therefore, the expression of the final results should be stated carefully. For example, in the VERTICAL trial, the interpretation of the results was expressed as: “we found no differences in pain response, pain scores, and global QOL between patients receiving cRT and those (offered to be) treated with SBRT” (p. , [36]). The part between brackets points out that treatment effects represent the effect of offered alternative treatment compared to receiving SOC rather than a comparison of patients receiving two different treatments [54]. The same phrasing with respect to the treatment effect under the ITT principle was adopted when presenting the UMBRELLA FIT trial results. In addition, for the UMBRELLA FIT trial, results were reported for patients offered the alternative intervention as well as those for patients accepting the alternative intervention [16].

Finally, analyzing a TwiCs study according to the treatment policy strategy ensures that the occurrence of the intercurrent event is also of main interest [56], which means that a TwiCs study can be used to gain insight in the acceptability of an alternative treatment. This was recognized in the VERTICAL trial, where this acceptability was explicitly stated when discussing the results [36]. Therefore, acceptability of the alternative treatment could be part of the research question and must be seen as part of the treatment effect [57].

#### Principal stratum strategy

In addition to the treatment policy strategy, the ICH E9(R1) guideline lists four other strategies to address the research question. Each of these strategies approaches a different research question. We will briefly discuss one other strategy that plays a role in the TwiCs setting. This strategy is the principal stratum strategy where the intercurrent event is considered a confounding factor when estimating a treatment effect. In sum, the treatment effect is estimated in a (target) population (“stratum”) whose status with respect to the intercurrent event is similar, irrespective of treatment arm. For a TwiCs study, this means that the treatment effect is estimated in a population that is capable and willing to accept the treatment being assigned to. Using different analysis strategies than the ITT approach, an estimate of the treatment effect under perfect compliance can be generated, typically based on causal inference models [58]. An example of such an estimate is the complier average causal effect (CACE), which provides an unbiased treatment effect for patients who comply with the protocol [59]. This definition diverges from the ITT definition in a TwiCs study, which demonstrates that both estimands are concerned with a different research question.

The remaining strategies listed in the ICH E9 (R1) may also apply to the TwiCs design, but the treatment policy strategy and the principal stratum strategy have been described in publications of TwiCs trials, which is why we limit the discussion to these two strategies. For a detailed overview on how to define the estimand based on difference strategies with detailed examples, see [56, 60, 61].

In sum, different research questions can in principle be addressed by a TwiCs study. The research question drives the definition of the estimand(s) of interest in a TwiCs study, which should be defined before the start of the study. These definitions will then determine the primary analysis and, importantly, power and sample size assessment. It is crucial to mention that these different estimands should not be interpreted as alternatives to one another, but merely as ways to answer different research questions.

### Analysis of a TwiCs study

The effect of the alternative treatment arm compared to control can simply be estimated by comparing the group of patients randomized to the alternative treatment arm with the group of patients randomized to SOC, using an appropriate statistical test. This approach is similar to the primary analysis strategy of most randomized trials. However, the result of this analysis in a TwiCs study should not be interpreted as the ACE observed in a standard RCT, because the non-compliance rate observed in the intervention arm dilutes this effect and should be taken into consideration when interpreting the results.

When the main focus is the effect of the intervention under compliance (principal stratum strategy), the analysis must be adapted accordingly. In the TwiCs literature, instrumental variable (IV) analyses have been proposed to accomplish this [57, 62, 63]. These IV analyses use a two-stage least squares method to account for possible non-compliance in the alternative intervention group [64]. In the first stage, the effect of exposure (actual treatment received) is predicted by the effect of randomization. In the second stage, this information is used to understand how the exposure affects the outcome. Two different IV analyses were proposed by Pate et al*.* [63] and Candlish et al*.* [62] to analyze TwiCs studies. In the first IV analysis, a two-stage regression model is applied. In the first stage, the effect of randomization on exposure is estimated using logistic regression, which provides the estimated exposure given the allocated treatment. Subsequently, in the second stage, a regression model for the outcome is fitted using the estimated exposure from the previous logistic regression model as covariate. The effect of the estimated exposure on the outcome provides the estimated treatment effect of interest. The second IV analysis also starts with a logistic regression model predicting exposure by randomization, but here the residual term is calculated as the difference between actual exposure and predicted exposure. In the second regression model, the outcome is modeled as a function of the treatment received and the residuals calculated from the previous logistic regression where the coefficient of treatment received provides the estimated treatment effect.

In two simulation studies, the performance and accuracy of the ITT and IV analysis in analyzing TwiCs study results were investigated [62, 63]. The authors reveal that the larger the refusal rate, the more bias was found in the ITT effect as expected in a standard RCT. However, considering our arguments in the previous Section, this is a logical finding. When acknowledging that a TwiCs study estimates a different ITT effect compared to a standard RCT, it is expected that the ITT effect of a TwiCs study deviates from a (simulated) ITT effect of a standard RCT, but that should not be interpreted as bias. Again, bias in the ITT effect of a TwiCs study should not be seen as bias relative to the ITT effect of a standard RCT, but relative to its own definition. For example, when non-compliance depends on certain patient characteristics (*e.g.* only male participants refuse treatment), we can expect bias in the ACE of offered treatment relative to the population value. Furthermore, in the same simulation studies, it was also found that when refusal in the intervention arm is present, the IV analyses in a TwiCs study provided an effect estimate that was closer to the ITT effect estimate of a standard RCT than the ITT effect of a TwiCs study was to the ITT effect estimate of a standard RCT [62, 63]. This implies that for researchers who are interested in deriving a treatment effect from a TwiCs study that is close to the ITT of a standard RCT, IV analyses offer this possibility. However, this does not fix the issue of non-compliance and we believe that it is not necessary to fix this as long as researchers acknowledge that a TwiCs study estimates something different compared to a standard RCT.

With respect to the completed TwiCs oncology studies (Table 1), only the UMBRELLA FIT trial provided results of an ITT and IV analysis. In addition, in the UMBRELLA FIT trial, another alternative analysis strategy was used, namely a propensity score analysis by comparing intervention accepters to patients in the control group who would have accepted the alternative intervention if offered [16]. This propensity score analysis serves as a sensitivity analysis to the IV analysis, because it is unknown whether intervention refusers are influenced by the offer of the intervention.

### Statistical power

In general, sample size calculations should be based on the anticipated treatment effect according to the ITT definition. The anticipated ITT effect of a TwiCs study reflects the ITT effect considering non-compliance in the alternative treatment arm (offered treatment) and will therefore be smaller than the ITT effect in a standard RCT. As a result, required sample sizes for obtaining sufficient power in a TwiCs study are often larger than those of standard RCTs [62, 63].

A critical issue in TwiCs studies is that the expected non-compliance rate may diverge from the actual non-compliance rate, which was the case in the UMBRELLA FIT trial, the RECTAL BOOST trial, and the VERTICAL trial (see the Section on ‘Non-compliance in the alternative treatment arm’). Consequently, the sample size had to be updated during the trial based on the actual non-compliance rate, which was also recommended by Candlish et al. [62]. This can have severe implications when the observational cohort is limited in the number of available patients, which can be the case in a closed cohort [50]. Updating the required sample size is easier in recruiting cohorts. Furthermore, recruiting cohorts have the advantage that the non-compliance rate can be updated after each randomization and the sample size can be adapted until the actual non-compliance rate is reached. It has been recommended to calculate the required sample size under different non-compliance rate assumptions during the design stage [50, 54] or to first perform a pilot study before the actual TwiCs study to obtain insights in the actual refusal rates [17].

As a final note on the sample size we would like to point out that the discussion of the (diluted) ITT effect so far holds for superiority trials. A diluted ITT effect makes it easier to demonstrate non-inferiority or equivalence. In general, the ITT effect in non-inferiority trials is anti-conservative [65]. Therefore, in designing and analyzing TwiCs non-inferiority trials, a per protocol analysis excluding non-compliance should be considered. However, since non-compliance only occurs in the alternative treatment arm, it is unclear how this will affect treatment group balance and hence the interpretation of non-inferiority. To our knowledge, there have been no proposed or conducted non-inferiority TwiCs studies to date.

### Multiple TwiCs studies within the same cohort

Until now, the discussion about the methodological challenges encountered in TwiCs studies was focused on performing only one TwiCs study within a cohort. However, in the Introduction Section, we mentioned the possibility of running multiple TwiCs studies within the same cohort. A TwiCs study uses a broad prospective observational cohort study and this cohort typically represents a broad population of interest. When running multiple TwiCs studies within the same cohort, either consecutively or in parallel, these studies are most often considered separate, stand-alone trials that each answer a different research question and that use their own concurrent control and intervention participants. They may also target different sub populations within the cohort. This is not any different than performing multiple standard RCTs in a general population, or in collaborative networks across study sites and it is therefore not required to adjust for multiplicity or Type I error inflation when running multiple TwiCs studies within the same cohort. Only in the scenario where, e.g., different TwiCs studies use a shared control group, similar to how controls can be used in platform trials, a multiplicity correction (e.g. controlling the family wise error rate) may be required [66, 67].

Simulations have shown that when two TwiCs studies share the same control group, results between the two trials are correlated [68]. However, since the objectives of each individual TwiCs study stands on its own, there is no intention to investigate the effect of a series of treatments that are linked together. The scenario of overlapping control arms is thus not likely to occur. Also confounding between two treatment arms (control or intervention) of two different TwiCs studies (*e.g.* when patients can only receive the alternative treatment in one study) tend to result in correlated trial results [68], but this scenario is not very likely at all as it violates the equal treatment assignment probability across patients [69]. Moreover, observational cohort studies include a large number of patients and most cohort studies are recruiting cohorts, which also decreases the chance of overlapping treatment arms across TwiCs studies.

In sum, overlapping treatment arms across multiple TwiCs studies is considered a minor potential methodological challenge. However, if it does occur, the availability of a cohort study offers an important advantage, because a patients’ treatment status in other TwiCs studies within the same cohort is known and can thus be taken into account when randomizing patients for a new TwiCs study. For example, in the Dutch PLCRC, the RECTAL BOOST [28] and SPONGE [34] trial are two consecutive trials and the trial status of the RECTAL BOOST trial was used as stratification factor when randomizing patients for the SPONGE trial. In contrast, when running multiple standard RCTs within a general population, other trial inclusions are not structurally collected and therefore not always known.

## Discussion

This article provides an overview of examples of TwiCs studies conducted in the oncology setting, where the TwiCs design has gained increasing popularity during the last decade, especially in the Netherlands. The rise in initiated and conducted TwiCs studies is associated with several drawbacks that are encountered when performing standard RCTs for which the TwiCs study design offers possible solutions. These drawbacks are the risk of uncompleted trials partly due to slow and difficult accrual of patients, limited external validity in standard RCTs and high drop-out rates in the control group. Whether the latter is an issue in standard oncology RCTs can be questioned, since oncology patients already receive the best SOC and a study treatment is not commonly available (except for supportive care setting).

The main elements of a TwiCs study are that these are conducted within an observational cohort study, that patients which are randomized to the SOC receive no information about the trial and that patients randomized to the alternative treatment arm can refuse this treatment after randomization. The advantage of an available observational cohort is that patients can be found and contacted easily. This has been observed in two oncology trials where they found that accrual was faster compared to standard RCTs [17, 50]. Furthermore, another possible advantage of the availability of an observational cohort is that the collection of routine cohort measurements may minimize selection bias if eligibility is based on these measurements and the effect of selection by a physician is decreased [17]. At the same time, it is crucial to capture sufficient key demographics and other historical, disease specific variables at baseline upon cohort enrollment and that this information is regularly updated to determine eligibility for future TwiCs studies. Minimization of selection bias will likely improve external validity. Moreover, the fact that control patients are not informed will improve external validity, as this design element more closely resembles clinical practice.

Despite the possible advantages of a TwiCs study, the design is entailed with several methodological challenges that are addressed in this article. One challenge that is discussed here is the timing of randomization and the associated possible risk of selection bias for cohort enrollment. Another important challenge is the anticipated non-compliance (refusal) rate in the alternative treatment arm and how this non-compliance rate affects the definition of the estimand, the related research question, the analysis methods and the sample size calculation. It is important to emphasize that the ITT effect of a TwiCs study has a different meaning than the ITT effect of a standard RCT. The ITT effect of a TwiCs study estimates the ACE of offered treatment, whereas the ITT effect of a standard RCT estimates the ACE of received treatment. Consequently, the two ITT estimates are different by definition and the difference between the two estimates should not be considered bias, but merely as two estimates that represent answers to two different research questions. The unavoidable non-compliance in a TwiCs study also means that not all interventions are suitable for TwiCs study designs. Especially in situations when there are many safety issues or many required hospital visits involved, the risk of non-compliance may be too large. Therefore, researchers should carefully consider whether an intervention is suited for a TwiCs study.

An important question related to the ITT definition in a TwiCs study is whether the results of a TwiCs study could be accepted by regulatory authorities for approval of a new treatment. For pivotal trials, a TwiCs study leads to a loss of some benefits relative to a standard RCT. However, the loss of benefits is not primarily related to the fact that a TwiCs study provides no effect of received treatment, since a more conservative treatment effect estimate—the effect of offered treatment, may not necessarily be an issue for regulatory authorities. A main concern for regulatory authorities and reimbursement policies may be related to the fact that the population of interest for which a treatment has a positive benefit-risk is difficult to determine a-priori when the two treatment arms provide non-matching safety data due to non-random refusal across treatment arms.

The challenges faced in standard RCTs described at the beginning of this article might motivate researchers to conduct single-arm trials where external, historical controls can be used for comparison purposes. However, the lack of randomization in single-arm trials can cause serious bias and confounding and leads to difficulties in quantifying a treatment effect. Although methods have been proposed to adjust for confounding, bias, and imbalance, randomization is the only way to make detection of imbalances possible in the first place [70]. Therefore, although a TwiCs study is faced with methodological challenges and may not be preferred by regulatory authorities over standard RCTs, it can be considered a more suitable alternative compared to single-arm trials, because patients are randomized in a TwiCs study, thereby reducing sources of bias that are encountered in non-randomized trials. Notably, the choice of design in the oncology setting is dependent upon many factors. For example, ethical aspects play an important role in deciding whether randomization is feasible, as does the willingness of patients to be randomized. For a very thorough discussion and detailed overview of perspectives on the use of randomization in oncology trials, see Grayling et al*.* [71].

A final point of discussion is the validity of the assumptions stated in this article. We assumed that the occurrence of non-compliance (that is, refusal of assigned treatment as intercurrent event) only occurs in the alternative treatment arm, which is a valid assumption by nature of the design. However, this assumption is violated when patients randomized to SOC refuse to be treated with SOC. Refusing SOC is generally not very common in oncology (see for example three studies on the predictors associated with treatment refusal in colon [72], breast [73], and head and neck cancer [74], where small percentages of SOC refusal were reported). However, it cannot be ruled out. How to deal with this non-compliance in the control group in a TwiCs study context with respect to the analysis and interpretation of the results will depend on the research question and whether this control non-compliance will be judged as problematic. As such, researchers could determine a-priori whether this is likely to occur and then alter, for example, the expected effect size. Moreover, the assumption of no contamination in the control group may be violated when these patients become aware of the availability of an alternative treatment, either due to communication with other patients in the waiting room, or due to finding information online as trials are prospectively registered. In such circumstances, control patients might take the initiative in asking their treating physician to receive the alternative treatment. This may cause potential (ethical) dilemmas for trial staff and possible cross-over of control patients when these patients are already randomized to SOC without knowing it. However, whether this is a substantial risk should be considered per trial.

## Conclusion

In this article we provided an overview of potential advantages of a TwiCs study compared to standard RCTs and we reflected upon the most important methodological challenges of a TwiCs study, based on experiences in oncology. Researchers in oncology and other areas should carefully consider these methodological challenges when planning to initiate a TwiCs study.

## Data Availability

Any materials used during this study are available from author R. Kessels upon request via r.kessels@nki.nl.

## Abbreviations

ACE: Average causal effect
ITT: Intention-to-treat
IV: Instrumental variable
PLCRC: Prospective Dutch colorectal cancer cohort
PICNIC: Prospective data collection initiative in colorectal cancer
PRESENT: Prospective evaluation of interventional studies on bone metastases
RCT: Randomized controlled trial
SOC: Standard of care
TwiCs: Trial within cohorts study
UMBRELLA: Utrecht cohort of multiple breast cancer intervention studies and long-term evaluation
